# Symbiotic N_**2**_-Fixation Estimated by the ^**15**^N Tracer Technique and Growth of* Pueraria phaseoloides* (Roxb.) Benth. Inoculated with* Bradyrhizobium* Strain in Field Conditions

**DOI:** 10.1155/2016/7026859

**Published:** 2016-01-24

**Authors:** Papa Saliou Sarr, Judith Wase Okon, Didier Aime Boyogueno Begoude, Shigeru Araki, Zachée Ambang, Makoto Shibata, Shinya Funakawa

**Affiliations:** ^1^Center for African Area Studies, Graduate School of Asian and African Area Studies, Kyoto University, 46 Shimoadachi-cho, Yoshida, Sakyo-ku, Kyoto 606-8501, Japan; ^2^Department of Plant Biology, Faculty of Science, University of Yaoundé I, P.O. Box 812, Yaoundé, Cameroon; ^3^Regional Biocontrol and Applied Microbiology Laboratory, Institute of Agricultural Research for Development (IRAD), P.O. Box 2067, Yaoundé, Cameroon; ^4^Graduate School of Agriculture, Soil Science Laboratory, Kyoto University, Kitashirakawa Oiwake-cho, Sakyo-ku, Kyoto 606-8502, Japan

## Abstract

This field experiment was established in Eastern Cameroon to examine the effect of selected rhizobial inoculation on N_2_-fixation and growth of* Pueraria phaseoloides*. Treatments consisted of noninoculated and* Bradyrhizobium yuanmingense* S3-4-inoculated* Pueraria* with three replications each.* Ipomoea batatas* as a non-N_2_-fixing reference was interspersed in each* Pueraria* plot. All the twelve plots received 2 gN/m^2^ of ^15^N ammonium sulfate 10% atom excess. At harvest, dry matter yields and the nitrogen derived from atmospheric N_2_-fixation (%Ndfa) of inoculated* Pueraria* were significantly (*P* < 0.05) higher (81% and 10.83%, resp.) than those of noninoculated* Pueraria*. The inoculation enhanced nodule dry weight 2.44-fold. Consequently, the harvested N significantly (*P* < 0.05) increased by 83% in inoculated* Pueraria*, resulting from the increase in N_2_-fixation and soil N uptake. A loss of 55 to 60% of the N fertilizer was reported, and 36 to 40% of it was immobilized in soil. Here, we demonstrated that both N_2_-fixing potential of* P. phaseoloides* and soil N uptake are improved through field inoculations using efficient bradyrhizobial species. In practice, the inoculation contributes to maximize N input in soils by the cover crop's biomass and represent a good strategy to improve soil fertility for subsequent cultivation.

## 1. Introduction

The genus* Pueraria* originates from South Asia, Southeast Asia, China, Japan, and parts of Oceania [1] and is now widespread throughout the humid tropics. Perennial legume cover crops such as* Pueraria phaseoloides* are commonly grown between the rows trees or used in improved fallows, in order to reduce soil erosion, improve soil fertility, and increase the growth and yield of associated plants [2–4]. Leguminous crops are a source of nitrogen and contribute to an increase in the nitrogen uptake of the nonleguminous associated crop [5]. A major advantage of growing a legume cover crop is its ability to fix N_2_ symbiotically and to accumulate nitrogen in the plantation agroecosystem.* P. phaseoloides* fixes nitrogen in symbiosis with* Bradyrhizobium* strains and is classified as “promiscuous ineffective” [6]. Nitrogen fixation is a process by which nitrogen in the atmosphere is converted into ammonium [7]. Both the plant and the rhizobia benefit from such a symbiotic relationship. Nitrogen is the most commonly deficient nutrient in many soils around the world and it is the most commonly supplied plant nutrient. However, the supply of nitrogen through fertilizers has severe environmental concerns. Atmospheric nitrogen fixed by the symbiotic association between soil bacteria (rhizobia) and crop legumes is likely to be in the order of 20 to 22 million tons of nitrogen each year [8].

In Eastern Cameroon, short-term* Pueraria* fallows of two years are commonly practiced to restore soil fertility for subsequent cultivation of cassava which is a staple food in the area. Yield increments of cassava are related to soil quality improvement which includes soil erosion limitation, increase of soil organic carbon and nitrogen following a good decomposition of* Pueraria* litters. The phylogenetic diversity of nitrogen-fixing bacteria in the root nodules of* Pueraria* in Eastern Cameroon was previously investigated [9]. Using molecular biology techniques, these authors identified three groups of* Bradyrhizobium* species corresponding to* B.* sp.,* B. yuanmingense*, and* B. elkanii* that nodulate* Pueraria phaseoloides*. By assessing the nitrogen fixation potential of these three groups using the Acetylene Reduction Activity (ARA) method, it was found that strains belonging to* B. yuanmingense* showed significant higher efficiency. The selection of appropriate rhizobial microsymbionts is becoming a complex procedure due to the fact that several legumes species can be nodulated by single rhizobia [10]. Yet the symbiotic association between the legumes and their microbial symbionts plays a significant role in agriculture worldwide by reducing ca. 100 million metric tons of atmospheric nitrogen saving US$ 8 billion/year on fertilizer N [11, 12]. The common approach to improving the symbiotic fixation of nitrogen and legume productivity, using superior or very effective exotic rhizobial strains as inoculants, often fails to achieve the desired responses [13, 14]. The failure has been attributed to the poor competitiveness of the introduced rhizobia [15], the nonspecificity of the bacteria, and the occurrence and resistance to stress of ineffective indigenous strains in soils [16]. To improve the N_2_-fixation and growth of* Pueraria*, the use of rhizobia, selected from indigenous community with capacity for nodulation and strong nitrogen-fixing activity, is believed to be an important agronomic approach.

*Pueraria phaseoloides* is used in the tropics as a forage and cover crop, but rhizobial inoculations of this legume in field conditions have not been widely tested. The importance of this work is to improve the nitrogen fixation potential and growth of* Pueraria* through field inoculations with effective strains of* Bradyrhizobium*, enabling enhancement of the input of nitrogen in the soil via biological process, to shorten the fallow period of* Pueraria* and put the land in faster use, as well as reduce environmental hazards that the use of chemical fertilizers to increase yields often poses. In this paper, we report on the effects of inoculating* Pueraria* with the selected efficient* B. yuanmingense *S3-4 strain [9] on its N_2_-fixation, dry biomass production, total N uptake, nitrogen fertilizer use efficiency, and soil fertility through a field experiment. Symbiotic nitrogen fixation was estimated using the ^15^N tracer technique and* Ipomoea batatas* (L.) Lam. was used as a nonfixing reference plant.

## 2. Materials and Methods

### 2.1. Study Site

The study was carried out in Andom village (5°15′N, 13°30′E) located in Eastern Cameroon, which is in the forest-savanna transition zone [17, 18]. The elevation of the plateau is 650–700 m a.s.l. and its basement consists of metamorphic rocks. The annual rainfall range of this area is 1350–1550 mm and the distribution of rainfall is bimodal according to field measurement carried out from 2008 to 2013 [19]. Rains begin in mid-March, a short dry season follows from mid-July to the end of August, and then the main rainy season extends from September to the middle of November. The mean annual temperature in the region is 25°C. The December–February period is the driest part of the year when the relative humidity may be higher than 60% [20]. The soil pH in the cultivated land varies from 4.82 to 5.17 with average levels of organic C, total N, and available P of 1.81%, 0.113%, and 4.57 ppm, respectively, and a C/N ratio of 15.91 [21]. Soils are classified as Typic Kandiudox according to USDA classification system [22]. The dominant forest vegetation is* Albizia zygia*, which dominates the tree layer. The dominant species in the savanna are the perennial grasses* Pennisetum setaceum*,* Imperata cylindrica*, and* Chromolaena odorata* [17, 18]. The experiment was set on a cleared natural fallow land previously dominated by* Chromolaena odorata*. There have been no planted legumes including* Pueraria*, and no history of rhizobial inoculation was observed in the site.

### 2.2. Experimental Design

**Figure 1:**
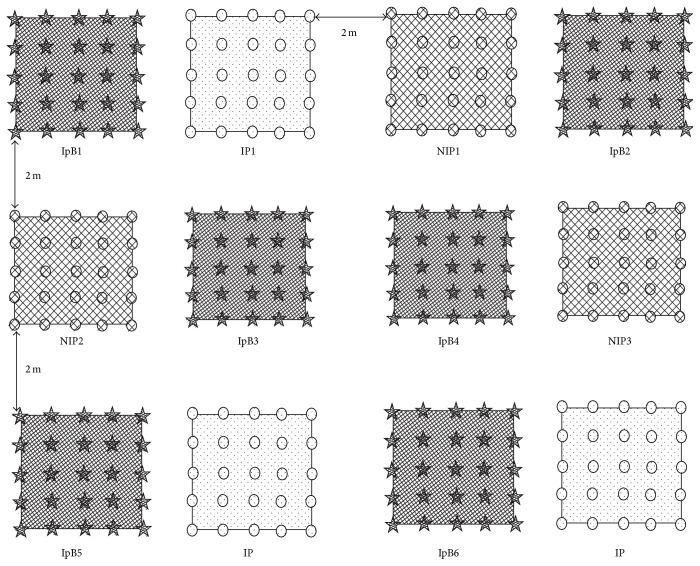
Experimental design. IP = inoculated* Pueraria*, NIP = noninoculated* Pueraria*, and IpB =* Ipomoea batatas*. IP and NIP treatments were each replicated 3 times, giving a total of 6* Pueraria* plots.* Ipomoea* plots (six) were interspersed in each* Pueraria* plot as non-N_2_-fixing references. Plot's size was 4 m^2^. In each plot, plant spacing was 50 cm corresponding to 9 hills per 1.5 m × 1.5 m for both* Pueraria* and* Ipomoea*. For* Pueraria*, seeds were sown but thinned to 3 plants per hill after the seedlings were well established while, for* Ipomoea*, uniform seedlings of 10 cm length were transplanted two weeks after the sowing of* Pueraria* seeds.* Pueraria* inoculation was performed using the effective strain* B. yuanmingense* S3-4 (2.094 × 10^8^ cells per hill) three weeks after seed's sowing. All plots received 2 gN/m^2^ of ^15^N labelled ammonium sulfate 10% atom excess applied at 1 and a half months after* Pueraria* seeds were sown. Plots were regularly weeded throughout the rain-fed prone experiment that lasted 4 months.

Treatments consisted of inoculated* Pueraria* and noninoculated* Pueraria*, each replicated three times in a complete randomized block design.* Ipomoea batatas* plots were interspersed in each* Pueraria* plot as non-N_2_-fixing references, giving a total of 12 plots (4 m^2^ plot^−1^) with a planting density of 9 plants per 1.5 m × 1.5 m.* I. batatas* was used as a reference crop as it shares with* Pueraria* similar patterns of N uptake from the soil and will obtain similar ^15^N enrichment in the soil-derived N [23]. The use of a reference crop is important in estimating the amount of nitrogen derived from the atmosphere (Ndfa) of legumes. Each* Pueraria*-*Ipomoea* block was separated to others by a 2 m interval (Figure 1).* Pueraria *seeds were sown on 9 August 2014 at 50 cm spacing. Uniform* Ipomoea* seedlings of 10 cm length were obtained from farmers' fields and transplanted in their corresponding plots at 50 cm spacing, two weeks after* Pueraria* seeds were sown.

### 2.3. Rhizobial Inoculation and Application of ^15^N Labelled Ammonium Sulfate

Prior to this field experiment, rhizobial strains were isolated from* Pueraria* nodules collected from the experimental site and other neighboring fields and subjected to DNA-based molecular analysis [9]. The results showed that the native strains for* Pueraria* in the experimental field clustered into 3 groups corresponding to* Bradyrhizobium yuanmingense*,* B. elkanii*, and* B. *sp. By assessing their nitrogen fixation potential using the Acetylene Reduction Assay (ARA), it appeared the* B. yuanmingense* strains, and S3-4 in particular, showed a significant highest potential. Therefore,* Bradyrhizobium yuanmingense* S3-4 identified as an efficient* Pueraria* symbiont [9] was used in this field experiment. From glycerol stock, it was streaked on YMA agar plates [24] for one week. After colony development, one pure colony was grown in 20 mL YMB for another week to produce the inoculum. The obtained solution of 2.094 × 10^9^ cells mL^−1^ was diluted 1000 times with sterilized water to give a concentration of 2.094 × 10^6^ cells mL^−1^. The inoculation was carried 3 weeks after the sowing of* Pueraria *seeds (29 August 2014) when seedlings were well established. Above each* Pueraria* hill (in the three inoculated* Pueraria* plots), 100 mL of the diluted inoculum corresponding to 2.094 × 10^8^ cells hill^−1^ was applied carefully. One and a half months after the starting of the experience when all crops were well established (22 September 2014) nitrogen fertilizer as ^15^N labelled ammonium sulfate enriched with 10% atom excess was applied at the rate of 2 gN/m^2^. When estimating the nitrogen fixation of legumes using labelled fertilizer N, lower rates of N fertilizer are preferably applied to avoid limiting the nitrogen fixation potential. Some authors [25, 26] also used the same rate of 2 gN/m^2^ during their studies on the N_2_-fixation of cowpea and* Pueraria* in field conditions. Prior to the fertilizer application,* Pueraria* seeds were thinned to three seedlings per hill. As 2 gN/m^2^ corresponds to 9.524 g of ammonium sulfate per square meter, the 12 plots in our experimental site required 475 g of ammonium sulfate. Considering the loss that may occur during the application of the fertilizer solution, we considered making a solution for 13 plots (495 g of ammonium sulfate). This amount was dissolved in 78 liters of water after which a 6-liter solution was carefully spread in each plot using a sprayer. Due to the high cost of labelled fertilizers, we limited the experimental unit to a 4 m^2^ area per plot. After the fertilizer solution was applied, a thin layer of soil was spread above the plots to mimic the atmospheric volatilization of the fertilizer. A portion of the solution was stored in a small bottle for further analysis of the exact atom percent excess of the fertilizer solution. Throughout the experimental period, plots were carefully and regularly weeded to avoid removing the ^15^N labelled ammonium sulfate outside the plots.

### 2.4. Data Collection

*P. phaseoloides* litter begins to accumulate on the soil surface about 6 months after establishment followed by subsequent mineralization [27]. To avoid entering the period of* Pueraria* leaves drop-off which would alter the results, this experiment lasted four months (August–December 2014). At term, the 9 central plants of each plot (to avoid the border effect) were harvested (Figure 1). The fresh shoots were separated from the fresh roots in each plot and their biomass was weighed.* Pueraria* root nodules were separated from the roots in each plot and their fresh weight was recorded. Fresh subsamples of known weights were collected from the shoot biomass and root biomass of each* Pueraria* and* Ipomoea* plot and together with the fresh nodules per* Pueraria* plot; they were oven-dried at 70°C for 48 hours in laboratory. Conversion calculations were made to determine the dry matter yields of inoculated* Pueraria*, noninoculated* Pueraria*, and* Ipomoea*. Composite soil samples were collected from each plot at the 0–15 cm horizon, air dried, and used for nutrient analysis.

The dried plant samples were ground at <0.5 mm and used for analysis. Total N was determined on the finely ground plant materials using 50 mg samples. The standard was 30 mg L-glutamic acid. Samples were packed in tin capsules and analyzed on a Carlo Erba NA1500 N/C elemental analyzer [28]. Total N (%) of soil samples was determined following the same procedure except that the amount of 500 mg was used. Total carbon was determined using a dry-combustion method with a C/N analyzer (VarioMax CHN; Elementar Analysensysteme GmbH, Hanau, Germany). The ^15^N enrichment in soil and plant materials was determined on an isotope ratio mass spectrometer (Delta Finnigan MAT 251, Bremen, Germany) coupled on-line to an elemental analyzer [28]. The ^15^N fertilizer solution used in this experiment was also analyzed to record the exact ^15^N atom% excess.

### 2.5. Calculations

The ^15^N atom% excess in plant materials was obtained by using the ^15^N atom% provided during the analysis and the atmospheric natural ^15^N abundance (0.3662%) by applying the equation

(1) N15  atom%  excess=N15  atom%−natural  N15  abundance.

In addition, the total nitrogen (TN; g/m^2^), fertilizer nitrogen recovery (g/m^2^), fertilizer nitrogen use efficiency (%NUE), the percentage of N crops that derived from the fertilizer (%Ndff), nitrogen derived from atmosphere (%Ndfa), and nitrogen derived from soil (%Ndfs) were determined.

Most calculations in this paper were according to the International Atomic Energy Agency. The fertilizer nitrogen recovery, which corresponds to the amount of nitrogen in plants that derived from the applied nitrogen (Ndff), was calculated as

(2) $$\begin{array}{c} Ndff\left(\;g/m^{2}\;\right)=\left(\;TN\times \frac{\%Ndff}{100}\;\right). \end{array}$$

The %Ndff was calculated using the following equation:

(3) %Ndff=N15  atom%  excess  of  plant  sampleN15  atom%  excess  of  fertilizer×100.

The TN and NUE were derived from the following calculations:

(4) $$\begin{array}{c} TN\left(\;g/m^{2}\;\right)=\left(\;\frac{DM\times N\%}{100}\;\right). \end{array}$$

DM (g/m^2^) corresponds to the dry matter yield

(5) $$\begin{array}{c} NUE\left(\;\%\;\right)=\left(\;\frac{Ndff\left(g/m^{2}\right)}{Applied\;\;N\left(g/m^{2}\right)}\;\right)\times 100. \end{array}$$

The proportion of* Pueraria* nitrogen derived from the atmosphere (%Ndfa) was calculated using the ^15^N isotope dilution equation [29]:

(6) $$\begin{array}{c} \%Ndfa=\left(\;1-\frac{{Ndff}_{fix}}{{Ndff}_{ref}}\;\right)\times 100. \end{array}$$

In (6), Ndff_fix_ is the %Ndff of the N_2_-fixing plant and Ndff_ref_ the %Ndff of the non-N_2_-fixing reference. As for* Pueraria*, the sources of nitrogen are the soil, the fertilizer, and the atmosphere; %Ndfs was calculated as

(7) $$\begin{array}{c} \%Ndfs=100-\%Ndff-\%Ndfa. \end{array}$$

For the non-N_2_-fixing* Ipomoea* reference crop,

(8) $$\begin{array}{c} \%Ndfs=100-\%Ndff. \end{array}$$

The TN (g/m^2^) and Ndff (g/m^2^) in roots, shoots, and nodules of* Pueraria* were determined separately. The sum of TN in shoots, roots, and nodules gave the total nitrogen content in the plant while the sum of Ndff in the three components yielded the total Ndff in the plant. For* Ipomoea*, the TN (g/m^2^) in roots and shoots were summed to give the plant total nitrogen, and similarly, the total nitrogen derived from the fertilizer was the sum of those in shoots and roots. These total values were used in the final calculations. To calculate the %Ndfa in a* Pueraria* plot (Ndfa of plant or Ndfa of residual soil) we considered the %Ndff (Ndfa of plant or Ndfa of residual soil) of the* Ipomoea* grown in interspersed plots.

The available amount of nitrogen in the soil [total soil N (g/m^2^)] in terms of equivalents units of ammonium sulfate was estimated in each plot. By using a labelled fertilizer source it is possible to determine the plant available amount of nutrient in the soil and this amount is expressed relatively to the amount available in the fertilizer source. Some authors [30–32] assumed that when a plant is confronted with two or more sources of a nutrient element, the nutrient uptake from each of these sources is proportional to the amounts available in each source. Implicit in this assumption is the fact that there is a complete mixture of the isotopes or isotopic equilibria. In this situation, the following equation was proposed:

(9) $$\begin{array}{c} \left(\;\frac{Plant\;\;Ndff}{Applied\;\;fertilizer\;\;N}\;\right)=\left(\;\frac{Plant\;\;Ndfs}{Total\;\;soil\;\;N}\;\right). \end{array}$$

Therefore,

(10) Total  soil  Ng/m2=Applied  fertilizer  N×plant  Ndfsplant  Ndff

N immobilization that corresponds to the portion of fertilizer N remaining in the soil at harvest is calculated as

(11) $$\begin{array}{c} N\;\;immobilization\left(\;\%\;\right)=\left(\;\frac{Soil\;\;Ndff}{Applied\;\;N}\;\right)\times 100. \end{array}$$

Following that, the amount of fertilizer N lost during the trial was calculated as follows:

(12) N  lossg/m2=applied  N−soil  Ndff+plant  Ndff.

Finally, N loss values can be transformed into percentages.

All data were subjected to analysis of variance (ANOVA) using the CropStat 7.2 software (International Rice Research Institute, Manila, Philippines) at a probability level of 5%. Mean separation was performed using Fisher's least significant difference (LSD) test whenever a significant result (*P* < 0.05) was obtained.

## 3. Results and Discussion

### 3.1. Growth Response of Plants to Bradyrhizobial Inoculation and Fertilizer Application

Fresh and dry matter yields of* Pueraria* and* Ipomoea* are reported in Table 1. It shows a positive effect (*P* < 0.05) of inoculation with* Bradyrhizobium yuanmingense* S3-4 on* P. phaseoloides* growth 4 months after seed sowing. Total fresh and dry weights were significantly higher in inoculated* Pueraria* than in noninoculated* Pueraria*. This difference originated from the difference in shoot production, given that no significant difference in root's yield was recorded between the inoculated and the noninoculated* Pueraria*. The inoculation contributed to increase the total fresh and dry weights of* Pueraria *2.02- and 1.81-fold, respectively. The 1.81-fold increase of the dry weight of* P. phaseoloides* receiving the inoculation was 81% higher compared to that of* Pueraria* without inoculation. The nodule fresh and dry weights were also significantly (*P* < 0.05) improved 2.32- and 2.44-fold by the inoculation, respectively. The increase in nodule dry weight from 0.283 g/m^2^ in the noninoculated* Pueraria* to 0.690 g/m^2^ in the inoculated* Pueraria* after 4-month growth (Table 1) could derive from the increase in nodule number or bigger nodule size. In this study, the nodule number was not counted at the field during the harvest due to high numbers but rather their total fresh and dry weights per plot were recorded. However, it is well known that matured bigger nodules with stronger hemoglobin activity are better drivers for increased N_2_-fixation in legume crops [33] rather than a high number of small immature nodules. It was found that the N_2_-fixation in groundnut was stimulated along with growth of nodules by application of another external source as millet straw [34]. Similarly,* Pueraria* plants with a greater mass of effective nodules tend to develop greater plant biomass.

In addition, although the main goal of this work was to compare the effect of the inoculation on* Pueraria* growth characteristics and N_2_-fixation, we observed that the dry matter production of the reference crop (*I. batatas*) was not significantly different from that of* Pueraria*.

**Figure 2:**
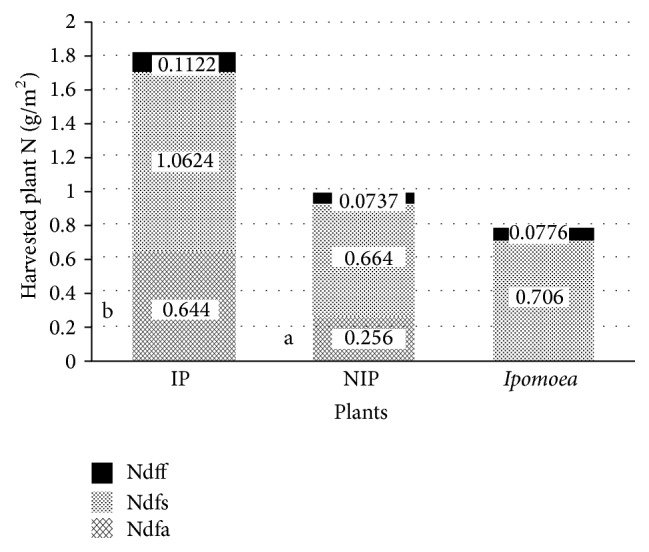
Sources and amount of plant N (g/m^2^) harvested per treatment during the four-month experiment period. Ndfs = nitrogen derived from soil, Ndff = nitrogen derived from fertilizer, and Ndfa = nitrogen derived from atmosphere. IP = inoculated* Pueraria*, NIP = noninoculated* Pueraria*. In a column, values correspond to the amount of N (g/m^2^) per N source (soil, fertilizer, and atmosphere). One-way ANOVA using CropStat ver. 7.2 was applied to the same N source for IP, NIP, and* Ipomoea* and no significant difference was observed except for Ndfa between IP and NIP with different letters.

Table 2 shows the nitrogen status in plants at harvest. Total N uptake in the reference crop (*Ipomoea*) was not significantly different from that of the noninoculated* Pueraria* but was significantly lower than that of the inoculated* Pueraria*. In addition, the harvested total N harvested from inoculated* Pueraria* was significantly higher than that harvested from noninoculated* Pueraria* at 5% probability level. The higher N content in inoculated* Pueraria* could be expected since it is strongly related to the higher dry matter production in this treatment. An overall increase of 0.825 g/m^2^ was obtained in inoculated plots within four months. To understand whether this increase in total N was a consequence of the obtained improvement of the N_2_-fixation itself, the total N in inoculated and noninoculated* Pueraria* plants was fractionated into nitrogen derived from soil (Ndfs), nitrogen derived from fertilizer (Ndff), and nitrogen derived from atmosphere (Ndfa), and that in* Ipomoea* was fractionated into Ndfs and Ndff, and results are shown in Figure 2. We observed that as a non-N_2_-fixing reference crop* Ipomoea* extracted most of its N from soil. The contribution of the N_2_-fixation in the plant's N pool was significantly higher (*P* < 0.05) in inoculated than in noninoculated* Pueraria*. The nitrogen derived from N_2_-fixation accounted for 26.25% in noninoculated* Pueraria* and 37.08% in inoculated* Pueraria *(Table 2). The results of plant N fractionation indicated that the surplus of 0.825 gN/m^2^ in the inoculated* Pueraria* did not derive only from the improvement of N_2_-nitrogen fixation. While +0.388 gN/m^2^ (47%) in inoculated* Pueraria* was added by the N_2_-fixation, the others, 0.398 gN/m^2^ (48.3%) and 0.039 gN/m^2^(4.61%), derived from the soil and applied fertilizer N, respectively. This clearly indicates that the inoculation of* Pueraria* with* B. yuanmingense* S3-4 not only enhances the N_2_-fixation but also may make a positive contribution in facilitation of a better uptake of N from the soil and from the applied fertilizer. For instance, Table 2 shows better fertilizer N use efficiency of the inoculated* Pueraria* than the noninoculated* Pueraria*. It is important to note that as the harvested plants were located in the middle of each plot, there was no risk of nutrient uptake from the external soil environment which would result in erratic data due to the dilution of the isotope. Furthermore, the interspersed design where each* Pueraria* plot was next to a plot of the reference* Ipomoea* reduced the fertility gradient that may exist in the experimental site.

The dual increase of N from N_2_-fixation and soil N uptake in inoculated* Pueraria* as shown in Figure 2 is not surprising and agrees with Butler and Ladd [35]. These authors also found that the amount of N_2_ fixed in medic (*Medicago littoralis*) and pea (*Pisum sativum*) increased together with increasing uptake of soil N in different soils when amounts of available N did not exceed 35 *µ*g N g^−1^ soil for medic and 74 *µ*g N g^−1^ soil for pea. The amount of soil N in the* Pueraria* plots of this study (Table 4) was higher (2.1 mg g^−1^ on average) than those values reported by Butler and Ladd [35]. However, Vesterager et al. [27] reported that apparently* P. phaseoloides* is tolerant towards the elevated inorganic N levels in the soil, which is an important quality for a cover crop in order to accumulate N in plantation systems. Moreover, by studying the effect of litter application on the N_2_-fixation of* Pueraria*, these authors found a higher amount of total N in the plants supplied with litter since the total uptake of soil N increased considerably by litter application. Similarly to their finding, the effect of the fertilizer N in our experiment was slightly improved by the bradyrhizobial inoculation as indicated by the %NUE and described above.

Table 3 shows the N status in shoots, roots, and nodules of inoculated* Pueraria*, noninoculated* Pueraria*, and* Ipomoea*. It indicates that the inoculation did not influence the N concentration in* P. phaseoloides*. Therefore, the 83% increase in total N of inoculated* Pueraria* compared to noninoculated* Pueraria* as shown in Table 2 was a result of the higher dry matter yield (Table 1) which resulted from a combination of (i) higher N_2_-fixation, (ii) soil N uptake, and (iii) a better use efficiency of the fertilizer N.

In the present study, the contribution of the symbiotic N_2_-fixation to the total N in* P. phaseoloides* (26–37%) was lower than that found by some authors in pot [27] and in field [23, 36] conditions which reached 70%.* P. phaseoloides* without inoculation was estimated by ^15^N isotope dilution to fix between 60 and 80% of the N it accumulated in a 2-year-old palm plantation in Malaysia, amounting to 151 kg N ha^−1^ year^−1^ [26]. Galiana et al. [37] inoculated* Acacia mangium* trees with two effective* Bradyrhizobium* sp. strains and estimated their nitrogen fixation by the ^15^N natural abundance method and reported that 55–62% of N derived from atmosphere in the inoculated trees while the noninoculated control trees fixed 41% of their nitrogen. The lower N_2_-fixation level in our experiment could partly be related to the lower plant density (9 plants/1.5 m × 1.5 m) we used for the purpose of the trial. The most adequate spacing for* Pueraria* for a fast soil cover was estimated to be 25 cm [38] which would result in a density of 25 plants/1.25 × 1.25 m. The shortage of rains during the first weeks of the experiment at the end of August 2014 (data not shown) that led to watering of the plants could be another reason. Furthermore, given that our experiment was set on a cleared natural fallow land, adequate N amount from the soil and the subsequent mineralization of residual litters may also have lowered the nitrogen fixation potential. High inorganic N concentrations are known to inhibit N_2_-fixation [39]. However, variation exists in the sensitivity of symbiotic N_2_-fixation to combined N between and within legume species [40, 41]. For instance, with* C. caeruleum*, no response to inoculation with several rhizobial strains was found in four experiments in Malaysia, even though the inoculant strains formed over 70% of the nodules in some cases [23]. In further experiments, inoculation of* C. caeruleum *and* Pueraria *with different strains of* Bradyrhizobium* also failed to improve their growth [42]. In some cases, the failure of cover crop establishment has been linked to the lack of suitable inoculants.

The ^15^N% atom excess in* Pueraria* (inoculated or noninoculated) shoots was found to be lower than that in* Ipomoea* (Table 3). This is to be expected since the N obtained by* Pueraria* from the soil and fixation from the atmosphere has reduced the ^14^N/^15^N ratio in the tissues [41] of the legume crop, contrary to the nonnodulating reference crop. Although it was not significant, the ^15^N enrichment in shoots, roots, and nodules of* Pueraria* slightly decreased by the bradyrhizobial inoculation, justifying the slight reduction of the ^14^N/^15^N ratio and increase in N_2_-fixation in these three plant components. In* P. phaseoloides*, the enrichment was higher in roots than in shoots and nodules. A similar result was obtained in a study of the symbiotic N_2_-fixation of* P. phaseoloides *as influenced by litter mineralization, using the grass* Axonopus compressus* as a non-N_2_-fixing reference crop [27]. This has been attributed to a relatively higher dilution of labelled ^15^N in shoots of legumes than in roots by symbiotically fixed N [35, 43]. Our results agree with Oghoghorie and Pate [44] who found that symbiotically fixed N accumulated in shoots and nodules whereas the roots derived most of its N from mineral sources. The pattern of higher enrichment in shoots than in roots in* I. batatas* might be a result of variations in the enrichment in available N with time. Such variations may be affected by soil type, plant species, and length of the growth period [27]. The total N in shoots, roots, and nodules reported in Table 3 indicates that rather than the roots and the nodules the shoots explain better the difference in total dry matter yield and total N harvest between the inoculated and the noninoculated* Pueraria*. For this reason many studies using the ^15^N enrichment technique focus on analyzing only the aboveground biomass.

### 3.2. Uptake of Fertilizer and Soil N

The recoveries of labelled fertilizer N (Ndff) in inoculated* Pueraria*, noninoculated* Pueraria*, and* Ipomoea* were 6.16%, 7.78%, and 10.27%, respectively (Table 2). The percentage of fertilizer N recovered in the nonfixing* Ipomoea* crop was significantly higher than that in the legume crop. This is particularly important since non-N_2_-fixing references require more nitrogen from other sources such as applied fertilizer N compared to legumes that fix atmospheric nitrogen as another source of N. However, the recovery of labelled fertilizer N was very low compared to the results of other studies. It reached 63% for* P. phaseoloides* and 71% in the* A. compressus *reference [27]. However, the %Ndff in this study was similar to that obtained in cowpea grown in field conditions [25]. The low recovery of labelled fertilizer N in the present study which correlated with the low NUE (%) could be related to the high loss (55–60%) of labelled fertilizer N from the top 15 cm of soils (Table 4). Nitrogen can become unavailable to the crop by leaching or as gaseous losses due to denitrification and/or volatilization. The concept of fertilizer use efficiency implies not only the maximum uptake of the applied nutrient by the crop but also the availability of the applied nutrient under variable climatic and edaphic conditions. Although this study was carried out to determine the nitrogen fixation of* Pueraria* following field inoculations, it is important to study the efficient use of fertilizers in order to obtain the highest possible yield with a minimum fertilizer application. Moreover, 36 to 40% of the labelled fertilizer N was immobilized in the soil (Table 4). Immobilization refers to the incorporation of nitrate and exchangeable ammonium into organic forms that are not available to plants. This occurs as microorganisms compete with plants for available nitrogen, which results in a decrease in the nitrogen availability for the crop. The low recovery of the labelled fertilizer N justified the high proportions (%) of Ndfs in plant materials. It was significantly higher in the reference crop (89.73%) than in the inoculated (56.77%) and the noninoculated (66.92%)* Pueraria* as shown in Table 2. Although no significant difference was observed in the %Ndfs between the inoculated and noninoculated* Pueraria* biomass, there was an overall increase in the amount of N uptake (g/m^2^) from soil. This is in contradiction with other studies which demonstrated that plants tend to extract lesser nutrients from the soil stock when other sources are available. In this regard, Rees et al. [45] reported a reduced uptake of soil N following application of legume residues with a high N content. A reduction of the recovery of ^15^N labelled fertilizer by application of legume residues with a low C/N ratio was also reported [46]. Although the analysis was not significant at 5% probability level, the residual soil N mainly derived from the original soil solution, small N portions were recovered from the applied ammonium sulfate fertilizer but also from the N_2_ fixed and returned to the soil within the four-month growth.

## 4. Conclusion

The inoculation with an effective bradyrhizobial strain stimulated the growth of field grown* P. phaseoloides* and increased the total N amount via an increase of the amounts of N derived from symbiotic N_2_-fixation and from soil. The positive effect of the inoculation on the nodulation phenotype (increase of nodule dry weight) and N_2_-fixation of* P. phaseoloides* shown in this study indicates that the introduced* B. yuanmingense* S3-4 was competitive with indigenous strains in field conditions. However, to find out whether this improvement was mainly due to the inoculation, the identification of infection with the used strain in the nodules would provide more accurate information. This study is important in that it is the first report describing the nitrogen fixation potential and growth of inoculated* P. phaseoloides* in field conditions with an effective* Bradyrhizobium* strain in Eastern Cameroon. Furthermore, it showed the positive contribution of field inoculations in maximizing N input in soils during fallows, for subsequent cultivation. The subject of the study has some relevance to soil fertility management in Cameroon which is one of the limiting factors of agricultural production.

## Figures and Tables

**Table 1:** Growth characteristics of plants (inoculated and noninoculated *Pueraria*, *Ipomoea*) after 4-month cultivation.

Treatments	Fresh weight (g/m^2^)				Dry weight (g/m^2^)			
	Shoot	Root	Nodule	Total	Shoot	Root	Nodule	Total
I. *Pueraria*	303.3b	12.0	2.667b	318b	64.0b	03.6	0.690b	68.29b
NI. *Pueraria*	144.0a	07.0	1.148a	152a	35.1a	02.1	0.283a	37.48a
*Ipomoea*	179.7a	44.3	—	224ab	29.9a	12.3	—	42.20ab
Sign. Trait (*P*_0.05_)	*∗*	ns	*∗*	*∗*	*∗*	ns	*∗*	*∗*

I. *Pueraria* = inoculated *Pueraria*, NI. *Pueraria* = noninoculated *Pueraria*, Sign. Trait = significant trait at 5% probability level, Nod. mass = nodule mass, ^*∗*^significant at *P* < 0.05, and ns = nonsignificant. One-way ANOVA was performed using CropStat ver. 7.2.

In a column, values with different letters (a, b) are statistically different at the indicated significant trait.

**Table 2:** N status in plants (inoculated and noninoculated *Pueraria*, *Ipomoea*) at harvest.

Treatments	Total N	Ndff	Ndfs	Ndfa	NUE
	g/m^2^	%			
I. *Pueraria*	1.818b	06.16a	56.77a	37.09	5.61b
NI. *Pueraria*	0.993a	07.68a	66.92a	26.24	3.68a
*Ipomoea*	0.784a	10.27b	89.73b	—	3.88a
Sign. Trait (*P*_0.05_)	*∗∗*	*∗∗*	*∗∗*	ns	*∗*

I. *Pueraria* = inoculated *Pueraria*, NI. *Pueraria* = noninoculated *Pueraria*, Sign. Trait = significant trait at 5% probability level, and ns = nonsignificant; ^*∗*^significant at *P* < 0.05, ^*∗∗*^significant at *P* < 0.01; Ndff = nitrogen derived from fertilizer, Ndfs = nitrogen derived from soil, Ndfa = nitrogen derived from atmosphere, and NUE = fertilizer nitrogen use efficiency. One-way ANOVA was performed using CropStat ver. 7.2.

In a column, values with different letters (a, b) are statistically different at the indicated significant trait.

**Table 3:** Effect of bradyrhizobial inoculation on plant N status after 4-month growth.

Treatments	%N			^15^N atom% excess			Total N (g/m^2^)		
	Shoot	Root	Nodule	Shoot	Root	Nodule	Shoot	Root	Nodule
I. *Pueraria*	2.71	1.18b	4.8	0.615a	0.773	0.474	1.743b	0.042	0.033
NI. *Pueraria*	2.73	1.20b	4.3	0.742a	0.893	0.485	0.953a	0.027	0.013
*Ipomoea*	2.56	0.69a		0.995b	0.880		0.783a	0.084	
Sign. Trait (*P*_0.05_)	ns	*∗∗∗*	ns	*∗*	ns	ns	*∗*	ns	ns

I. *Pueraria* = inoculated *Pueraria*, NI. *Pueraria* = noninoculated *Pueraria*, Sign. Trait = significant trait at 5% probability level, and ns = nonsignificant; ^*∗*^significant at *P* < 0.05, ^*∗∗∗*^significant at *P* < 0.001. One-way ANOVA was performed using CropStat ver. 7.2.

In a column, values with different letters (a, b) are statistically different at the indicated significant trait.

**Table 4:** Soil N status in inoculated and noninoculated *Pueraria* and *Ipomoea* plots at harvest.

Treatments	OrgC	N	C/N	Total N	Ndfs		Ndfa		Ndff		N imb.	N loss	
	%			g/m^2^	%	g/m^2^	%	g/m^2^	%	g/m^2^	%	%	g/m^2^
I. *Pueraria*	3.08	0.212	14.52	18.33	91.77a	16.90	4.15	0.68	4.08	0.74	37.0	57.12	1.14
NI. *Pueraria*	3.00	0.209	14.33	17.88	94.80b	16.95	1.14	0.21	4.07	0.73	36.5	60.05	1.2
*Ipomoea*	2.98	0.214	13.95	19.55	95.84b	18.74	—	—	4.16	0.81	40.5	55.73	1.12
Sign. Trait (*P*_0.05_)	ns	ns	ns	ns	*∗*	ns	ns	ns	ns	ns	ns	ns	ns

I. *Pueraria* = inoculated *Pueraria*, NI. *Pueraria* = noninoculated *Pueraria*, Sign. Trait = significant trait at 5% probability level, and ns = nonsignificant; ^*∗*^significant at *P* < 0.05. Composite soil samples were collected from each plot at harvest and subjected to chemical analysis for organic carbon (OrgC; %), N (%), and ^15^N atom% excess using the methods described in the text. Following that, (3), (6), (7), or (8) in the text was used to calculate soil Ndff (%), Ndfa (%), and Ndfs (%). Soil total N is the average N in treatments at the end of the trial and has three origins (Ndfs = soil nitrogen derived from soil, Ndfa = soil nitrogen derived from atmosphere, and Ndff = soil nitrogen derived from fertilizer). Total N is calculated based on (10) in the text. N imb. = N immobilization (it corresponds to the percentage of the fertilizer N that remains in the soil at the end of the experiment out of the initially applied 2 g/m^2^; cf. (11)). One-way ANOVA was performed using CropStat ver. 7.2. N loss is the fertilizer nitrogen lost in the system during the experiment via denitrification or leaching or volatilization.

In a column, values with different letters (a, b) are statistically different at the indicated significant trait.
